# Transition Metal-Doped Layered Iron Vanadate (FeV_3-x_M_x_O_9_.2.6H_2_O, M = Co, Mn, Ni, and Zn) for Enhanced Energy Storage Properties

**DOI:** 10.3390/nano14211765

**Published:** 2024-11-03

**Authors:** Mawuse Amedzo-Adore, Jeong In Han

**Affiliations:** Department of Chemical and Biochemical Engineering, Dongguk University, Seoul 04620, Republic of Korea; mendeladore@dongguk.edu

**Keywords:** iron vanadate, transition metal, doped, interlayer spacing, supercapacitor

## Abstract

With its distinctive multiple electrochemical reaction, iron vanadate (FeV_3_O_9_.2.6H_2_O) is considered as a promising electrode material for energy storage. However, it has a relatively low practical specific capacitance. Therefore, using the low temperature sol–gel synthesis process, transition metal doping was used to enhance the electrochemical performance of layered structured FeV_3_O_9_.2.6H_2_O (FVO). According to this study, FVO doped with transition metals with larger interlayer spacing exhibited superior electrochemical performance than undoped FVO. The Mn-doped FVO electrode showed the highest specific capacitance and retention of 143 Fg^−1^ and 87%, respectively, while the undoped FVO showed 78 Fg^−1^ and 54%.

## 1. Introduction

Population growth coupled with technological advances to achieve an industrialized society has led to increasing energy demands, thereby putting pressure on the already depleted fossil fuel and other non-renewable energy sources. Renewable energy sources, such as wind, solar, and geothermal energies, among others, have been developed over the years to lessen the over-dependence on fossil fuels as well as their environmental concerns [1,2]. Despite the successes of renewable energy sources over the years, they are unreliable due to natural intermittent fluctuations, which hinder their usage when connected to electric grids. In view of this, electrochemical energy storage systems such as batteries and supercapacitors are considered effective systems to mitigate the challenges and effectively utilize renewable energy sources [3]. Compared to other electrochemical energy storage systems, Supercapacitors have long life cycle, high specific capacitance and high power density, as well as being environmentally friendly. This makes supercapacitors promising potential energy storage systems and its utilization has been seen in various applications [4,5,6,7,8].

Of much interest recently are binary metal oxides, due to their multiple oxidation states and high electrical conductivity, which results in superior electrochemical performance compared to their corresponding single-metal oxides. Among the binary oxides, metal vanadates, with the general formula M_x_V_y_O_z_, are considered a promising material for supercapacitor applicationsdue to their layered structure, synergistic effect between vanadium and transition metal, excellent kinetics, and high capacity [9,10,11,12]. One of such materials is iron vanadate, which exists in various phases (Navajoite, Fervanite, and Kazakhstanite) [13], where the Kazakhstanite phase (FeV_3_O_9_.2.6H_2_O or Fe_5_V_15_O_39_(OH)_9_.9H_2_O) with comparatively larger interlayer spacing and layered structure has caught the attention of researchers and has seen its usage in a range of applications [14,15,16,17,18]. In recent report, Mawuse et al. obtained layered iron vanadate phosphate with lesser water content (FeV_3_O_9_.2.1H_2_O) compared to the theoretical water content (FeV_3_O_9_.2.6H_2_O) via the low temperature sol–gel method. The material was successfully used as an electrode material for two-electrode symmetric supercapacitor application in an aqueous electrolyte [19]. However, enhancing its specific capacitance as positive electrode material in aqueous supercapacitance application is desirable.

Up until now, a great deal of work has gone into improving the ionic and electronic conductivity, suppressing side reactions, and reducing surface degradations of energy conversion materials [20,21,22,23]. Transition metal doping is considered one of the efficient methods for enhancing the intrinsic electrical characteristics of transition metal oxides among other electrochemical properties [24,25,26]. Additionally, the doped element enhanced structural stability by acting as a pillar to prevent lattice collapse and structural distortion, suppressing unwanted electrochemical side reactions, thereby improving the rate performance and cycle stability [27,28,29]. Three-dimensional transition metal interstitial doping has been proposed as an efficient means of improving the spintronic (electronic and magnetic) properties of VI_3_ monolayers based on first-principles calculations [30]. Chu et al.’s investigation revealed that Nb doping is capable of effectively stabilizing the structure of Ni-rich NCM at high voltage while suppressing the Li/Ni disordering, which enhances electrochemical performance [31]. Also, Jian et al. by DFT calculation and experimental methods demonstrated that Co/Zn co-doped NCNF exhibited improved catalytic activity with superior power density of 0.603 Wcm^−3^, compared to their mono-doped (Co or Zn) samples, by reducing the dissociative barrier of the –OOH intermediate during ORR [32]. Additionally, compared to pristine Mn_3_O_4_, transition metal-doped Mn_3_O_4_ nanocrystals have been found to exhibit a high specific capacitance and superior cycling stability [26,33]. Additional reports provided evidence of the role of transition metal doping in transition metal oxides to improve their electrochemical performances [34,35]. 

To the best of our knowledge, there is no report on the impact of metal doping on layered iron vanadate’s electrochemical performance. The purpose of this research is to examine the effect of transition metal doping on enhancing the electrochemical performance of iron vanadate in an aqueous supercapacitor.

## 2. Materials and Methods

### 2.1. Materials and Synthesis

The sol–gel method was used to synthesize individual transition metal-doped Fe(V_2_M)O_9_.2.6H_2_O (M = Co, Mn, Ni and Zn) as reported in a previous report [19]. All reagents used in this work were analytical grade. In a typical experiment, 2 mmol NH_4_VO_3_ and 1mmol of individual transition metal precursors [Co(NO_3_)_2_.6H_2_O, Mn(NO_3_)_2_.4H_2_O, Ni(NO_3_)_2_.6H_2_O and Zn(NO_3_)_2_.6H_2_O] were dissolved in 100 mL D.I. water and stirred at 90 °C for 1 h. 0.1 M Fe(NO_3_)_3_.9H_2_O solution was subsequently added drop-wise to the solution under stirring and kept for l hour at 90 °C. After cooling, the precipitate was collected and washed several times by centrifugation and dried overnight at 80 °C in air to obtain various transition metal-doped Fe(V_2_M)O_9_.2.6H_2_O (M = Co, Mn, Ni and Zn) henceforth referred to as Co, Mn, Ni, and Zn. Pristine FeV_3_O_9_.2.6H_2_O was also synthesized and, thus, referred to as FVO.

### 2.2. Characterization

X-ray diffraction (XRD) was used to identify the structural phase of the materials using a laboratory Rigaku Ultima V diffractometer (Bruker AXS, Karlsruhe, Germany) with Cu Kα radiation source. Field-emission scanning electron microscopy (FE-SEM, JOEL-7800F, Japan Electron Optics Laboratory, Tokyo, Japan) and transmission electron microscopy (TEM, JEM-2100Plus, Japan Electron Optics Laboratory, Tokyo, Japan) were used to observe the morphology and elemental distribution in the material. The elemental composition of the sample was characterized usi-ng X-ray photoelectron spectroscopy (XPS, mono, K-alpha—Thermo UK system, Horsham, UK). Bond structure analysis was conducted by Fourier transform infrared spectroscopy (FT-IR, PerkinElmer, Beaconsfield, UK).

### 2.3. Electrochemical Test

Electrodes were prepared with FVO and its transition metal-doped samples, acetylene black and polyvinylidene fluoride (PVdF) (as an active material, conductive material, and binder, respectively) in a ratio of 7:2:1 was mixed with N-methyl-2-pyrrolidone (NMP) as a solvent to form a slurry. The slurry is then pasted on a nickel foam substrate and dried under a vacuum at 120 °C for 5 h to obtain the working electrode. The electrochemical properties (such as galvanostatic charge–discharge (GCD)), cyclic voltammetry (CV), and impedance spectroscopy (EIS) of the electrodes were obtained via a three-electrode system (Biologic SP-150 workstation, BioLogic, Paris, France) in 3M KOH aqueous electrolyte, where FVO and its transition metal-doped samples, Ag/AgCl electrode and platinum wire, served as a working reference and counter electrodes, respectively.

## 3. Results and Discussion

The photo of the synthesized samples displaying their respective colors is shown in Figure S1, where Mn-doped FVO shows a distinctive color that is completely different from the brown color of all other samples. According to the defect chemistry theory, an externally doped metal atom or ion is frequently the dominant component regulating the access to the material’s internal structure by substituting lattice atoms and generating distortions or defects [36]. Furthermore, it has been suggested that the dopant’s site of occupancy will affect the material’s characteristics; therefore, knowing the dopant’s site of occupancy is essential to understand the relationships between the properties and the electrochemical behaviors of the materials. According to Keyan et al. [37], the occupancy site preference of a dopant can be theoretically predicted based on their respective ionic radius and electronegativity, according the equation;

(1) $$D_{M}=\left(\frac{X_{D}-X_{M}}{X_{M}}\right)+(\frac{r_{D}-r_{M}}{r_{M}})$$

where D_M_ is the degree of deviation, X_D_ and r_D_ are electronegativity and radius of the dopant, and X_M_ and r_M_ are the electronegativity and radius of the substituted ion. For all the doped materials, as presented in Table 1, the degree of deviation for vanadium is less than that of iron, based on the respective ionic radius [38] and electronegativity [39] of the transition metals (Table S1). This suggests that the dopants will preferentially occupy the vanadium site in the FeV_3_O_9_.2.6H_2_O structure to form FeV_2_MO_9_.2.6H_2_O (M = Co, Mn, Ni and Zn).

**Figure 1:**
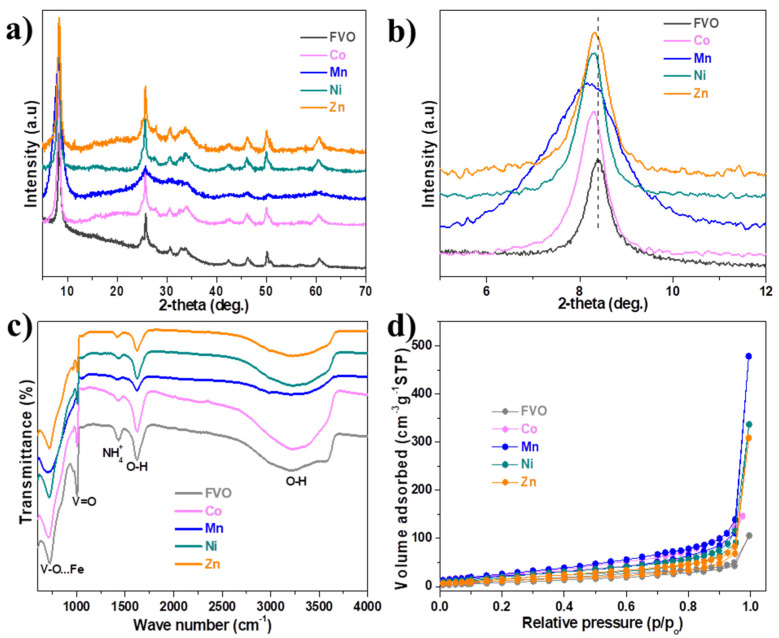
(**a**) PXRD patterns, (**b**) Expanded PXRD patterns, (**c**) FTIR spectra, and (**d**) N2 adsorption/desorption isotherm of undoped and doped FVO samples compared.

The powder XRD patterns of FeV_2_MO_9_.2.6H_2_O (M = Co, Mn, Ni, and Zn) samples are displayed in Figure 1a. All the samples’ diffraction peaks can be indexed to the single-phase FeV_3_O_9_.2.6H_2_O monoclinic structure, which has a space group C*/c-15 and lattice parameters a = 11.84 Å, b = 3.65 Å, and c = 21.27 Å, which are in agreement with the reported ICDC: 00-0-46-1334 [40,41]. All the doped samples show a modest shift in the diffraction peak at ~8.4° towards a lower angle (Figure 1b), indicating that the various transition metals have successful partially substituted V in the host lattice. This can be attributed to the larger ionic radii of the dopants (Co^2+^, Mn^2+^, Ni^2+^, and Zn^2+^) than V^4+^, as indicated in Table S1. As a result, the doped samples d-spacing increased, as shown in Table 2, which was determined using Bragg’s law of diffraction.

(2) $$d=\frac{nʎ}{2sinƟ}$$

The crystallite size of the samples is calculated from their respective full width at half maximum (FWHM) and the peak position of the most intense diffraction peak (~8.3°) by the Scherrer equation;

(3) $$D=\frac{k\lambda }{\beta cos\theta }$$

As summarized in Table 2, all the doped samples had smaller crystallite size than the pristine undoped sample. The Mn-doped FVO sample has the smallest crystallite size of 43.88 as opposed to 126.13 for pristine FVO. The bond structure of the pristine FVO remains unaffected by the doping of the transition metal, as shown by the FTIR study shown in Figure 1c. However, slight changes observed in the -OH bands (adsorbed water) could explain the fact that doping affects the amount of adsorbed water in the material.

Additionally, the N2 adsorption/desorption isotherm analysis (Figure 1d) demonstrates that the transition metal enhanced the specific surface area with the Mn-doped sample exhibiting the highest specific surface area of 88.71 m^2^g^−1^, compared to other samples, as shown in Table 3. The high surface area is essential to enhance ionic diffusivity, resulting in improved electrochemical performance.

**Figure 2:**
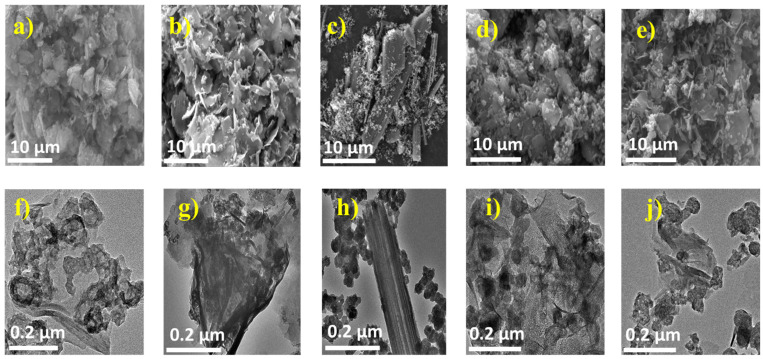
FE-SEM and TEM images of (**a**,**f**) FVO, (**b**,**g**) Co, (**c**,**h**) Mn, (**d**,**i**) Ni, and (**e**,**j**) Zn samples.

Figure 2 displays the samples’ FE-SEM and TEM images. The pristine FVO sample (Figure 2a,f) displayed a non-uniform plate-like morphology, whereas Co (Figure 2b,g), Ni (Figure 2d,i), and Zn (Figure 2e,j) samples showed a mixture of nanoparticles and nanosheets. However, the Mn-doped sample showed a mixture of nanoparticle and nanorod morphology. This unique morphology of the Mn sample is expected to improve electrochemical properties as it is expected to provide enhanced kinetic properties through 1D networks, as well as enhanced ionic diffusivity through direct contact between the electrolyte and particles [20,42].

**Figure 3:**
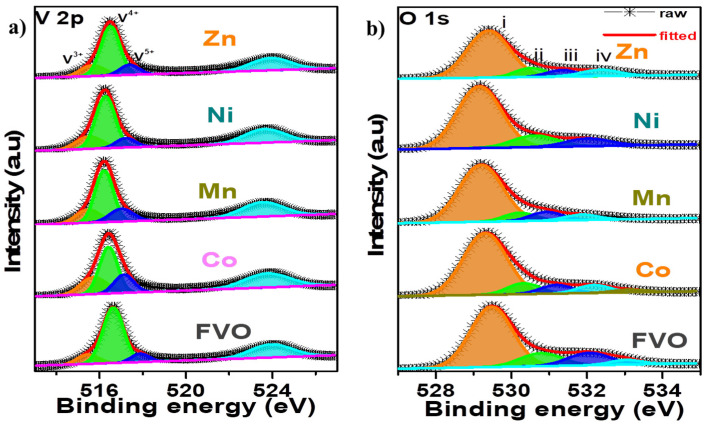
(**a**) V 2p spectra, and (**b**) O 1s spectra of all samples compared.

XPS measurements were employed to obtain the elemental compositions as well as to confirm successful transition metal doping of the samples. It is confirmed that all the samples contain a fundamental composition of Fe, V and O, as shown in Figure S2. The comparison of Fe 2p spectra of the samples (Figure S2a) shows no visible shift in their peak position, which indicates that the doped metal did not substitute the Fe site in the material. This is in agreement to the occupancy site preference of the dopants obtained via theoretical computation, as shown in Table 1. Furthermore, the deconvoluted V 2p spectra of all the samples were displayed in Figure 3a, where three major peaks assigned to V^3+^, V^4+^, and V^5+^ were obtained. However, careful comparison as illustrated in Figure S2b shows that, the metal-doped samples had lower binding energies ranging from ~516.49 eV to 516.19 eV, as compared to ~516.61 eV for pristine FVO sample. This phenomenon can be attributed to the reduction in the oxidation state of V as a result of charge compensation and balance of valence state. 

**Figure 4:**
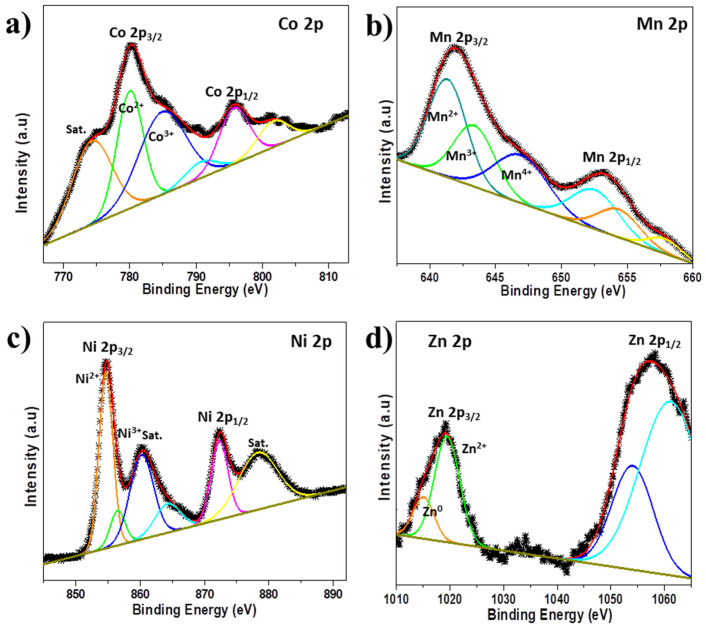
(**a**) Co 2p spectra of Co-doped FVO; (**b**) Mn 2p spectra of Mn-doped FVO; (**c**) Ni 2p spectra of Ni-doped FVO; and (**d**) Zn 2p spectra of Zn-doped FVO.

Additionally, the deconvoluted O 1s spectra of the samples were illustrated in Figure 3b, with four peaks assigned as (i) metal oxides, (ii) metal-hydrogen-oxide, (iii) surface adsorbed H_2_O, and (iv) chemisorbed or intercalated adsorbed H_2_O [19,43]. Further comparison of the O 1s spectra shows a shift to lower binding energy for all the doped materials compared to the pristine FVO sample (Figure S2c), which can be attributed to the formation of the dopant oxides, thereby confirming successful metal doping. Given that the dopant oxides have lower Gibbs formation energy than VO_2_, as indicated in Table S2 [38], it is anticipated that the successful doping to partially substitute V^4+^ in FeV_3_O_9_.2.6H_2_O will lower the bond strength. This phenomenon has the ability to decrease the interaction repulsion energy between potassium and potassium ions, which will enhance K^+^ diffusivity and improve electrochemical performances [44]. Furthermore, successful metal doping was confirmed by XPS, which shows Co, Mn, Ni, and Zn spectra, as shown in Figure 4.

**Figure 5:**
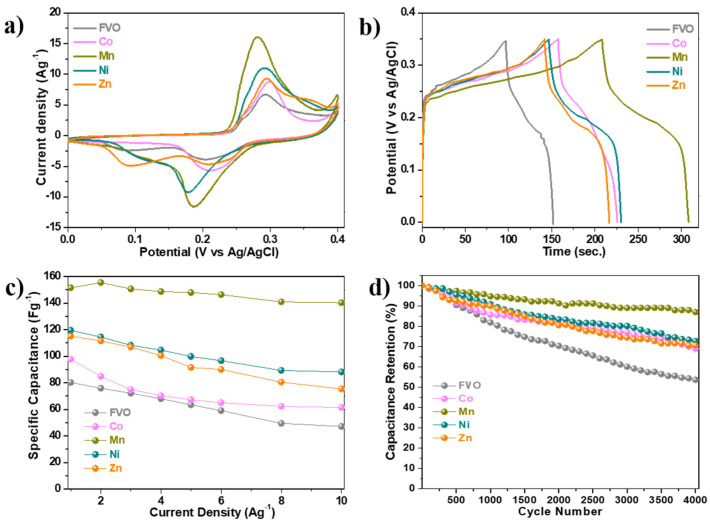
(**a**) CV curves at scan rate 2 mVs^−1^ in voltage window 0.0–0.4 V; (**b**) galvanostatic charge–discharge profiles measured at 0.5 Ag^−1^ in voltage window 0.0–0.35 V; (**c**) rate capability; and (**d**) cycle performances measured at current density 5 Ag^−1^; of all electrodes.

Using a three-electrode system in a 3 M KOH aqueous electrolyte, the effect of transition metal doping on the electrochemical properties of FeV_3_O_9_.2.6H_2_O was examined for supercapacitor application. The CV profiles of the electrodes at a scan rate of 20 mVs^−1^ and voltage window of 0.0–0.4 V are compared in Figure 5a, where all the samples showed identical anodic and cathodic peak shapes, indicating similar electrochemical reaction mechanism of characteristic pseudocapacitive behavior [19]. The metal-doped samples, however, have comparatively larger enclosed areas and peak currents. The Mn-doped sample exhibited the largest peak current, indicating that it has a higher capacitance than all other FVO samples. Figure S3a–e displays the CV profiles for pristine FVO and metal-doped (Co, Mn, Ni, and Zn) electrodes at different scan rates (10–50 mVs^−1^) and voltage windows 0–0.4 V. These profiles indicate strong reaction reversibility, which is demonstrated by well-maintained potential plateau shapes along with a characteristic peak shift with increasing scan rates. 

The galvanostatic charge–discharge curves of pristine and metal-doped FVO electrodes are compared in Figure 5b, measured at a voltage window of 0.0–0.35 V and current density of 0.5 Ag^−1^. The metal-doped FVO electrodes showed longer discharge times than that of pristine FVO, which translates into a specific capacitance of 97, 143, 119, and 107 Fg^−1^ for Co, Mn, Ni, and Zn, respectively, which are larger than 78 Fg^−1^ for pristine FVO. The rate capability of all the electrodes is presented in Figure 5c and their corresponding galvanostatic charge–discharge profiles are shown in Figure S4a–e. In comparison to pristine FVO, which had a specific capacitance of 47 Fg^−1^ and a capacitance retention of 58%, the metal-doped electrodes demonstrated improved rate performance, with high specific capacitance of 61, 140, 88, and 75 Fg^−1^, which corresponded to capacitance retention of 62.7, 92.6, 73.8, and 65.5%, respectively, at a high current density of 10 Ag^−1^ for Co-, Mn-, Ni-, and Zn-doped FVO, respectively. Furthermore, after 4000 cycles, the metal-doped FVO electrodes demonstrated enhanced cycle stability with capacitance retention of 69, 87, 73, and 71% for Co, Mn, Ni, and Zn electrodes, respectively. These are higher than 54% for pristine FVO, as shown in Figure 5d. The enhanced electrochemical performances of the transition metal-doped FVO electrodes can be ascribed to their larger d-spacing and shorter bond lengths, which increased ion diffusivity. This is further confirmed by the EIS analysis shown in Figure S5. 

**Figure 6:**
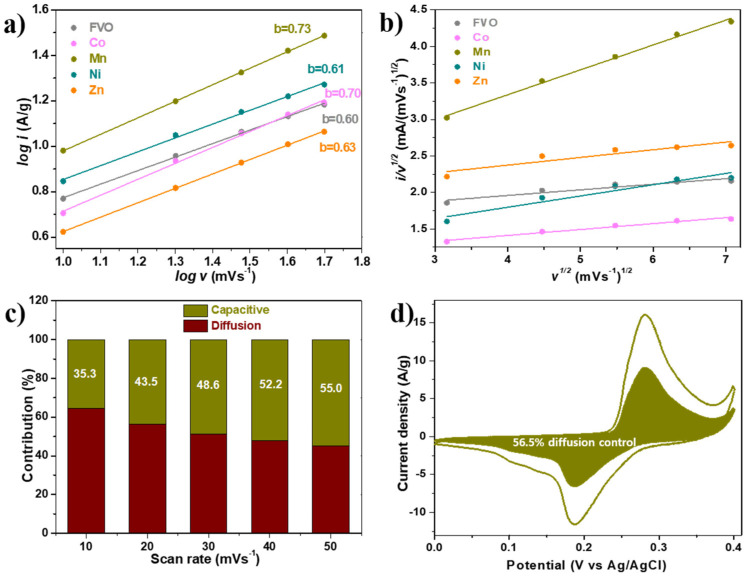
(**a**) Plot of log (v) vs. log (*i*) (b-value determination), and (**b**) plot of *i/v^1/2^* vs. *v^1/2^* of all electrodes compared. (**c**) Contribution ratio of surface-controlled process to the capacitance at various scan rates, and (**d**) the contribution ratio of the surface-controlled process to the capacitance at a scan rate of 20 mVs^−1^ for the Mn-doped FVO electrode.

Furthermore, the so-called b-value was evaluated using the power law relationship [43] to investigate the electrochemical reaction mechanism of the electrodes. As displayed in Figure 6a, all the electrodes have b-values indicating that their reactions are controlled by both surface and diffusion processes. Using Dunn’s method [43,45], the contribution ratio of the reaction mechanisms at various scan rates was further examined. 

The k1 and k2 values, which are shown in Table S3, were obtained from Figure 6b. It is observed that, regardless of the applied scan rate, the metal-doped FVO electrodes exhibited a higher contribution from the surface-controlled (capacitive) process than that of diffusion-controlled process as demonstrated in Figure 6c and Figure S6a–d. For instance, in Mn-doped FVO at a scan rate of 20 mVs^−1^, the surface-driven process contributes 43.5% to the capacitance, which is equivalent to 56.5% of the diffusion-driven process (Figure 6d).

## 4. Conclusions

FeV_3_O_9_.2.6H_2_O doped with different transition metals (M = Co, Mn, Ni, and Zn) was successfully synthesized using the sol–gel method. In comparison to the undoped FVO sample, the doped metal’s larger ionic sizes led to larger interlayer spacing and smaller crystallite sizes. To achieve charge neutrality in the material, the lower oxidation state of the dopants (M^2+^) led to a reduction in the oxidation state of V. Furthermore, as compared to the undoped FVO, the doped samples’ superior electrochemical properties are ascribed to the ionic diffusivity boosted by the reduced bond lengths attributed to lower Gibbs formation energies of the dopant transition metal oxides (M-O). Transition metal-doped FVO electrodes were shown to demonstrate superior electrochemical performances, with the highest specific capacitance of 143 Fg^−1^ recorded by the Mn-doped FVO electrode as compared to 78, 97, 119, and 107 Fg^−1^ for undoped FVO, Co, Ni, and Zn-doped electrodes, respectively. Additionally, with 87% capacitance retention, the Mn-doped FVO electrode demonstrated better rate performance and cycle stability than undoped FVO, Co-, Ni-, and Zn-doped FVO electrodes, which were 54, 69, 73, and 71%, respectively. The superior performance of Mn-doped FVO can be attributed to its larger d-spacing and lower crystallite size due to its 1D morphology.

It is believed that this work will pave the way for further research on doping in FeV_3_O_9_·2.6H_2_O samples for enhanced electrochemical performance in supercapacitor applications.

## Figures and Tables

**Table 1:** Degree of deviation of Fe and V for various transition metal dopants.

Degree of Deviation	Co^2+^	Mn^2+^	Ni^2+^	Zn^2+^
D_Fe_	−0.0061	−0.0144	0.0814	0.1340
D_V_	−0.1668	−0.1714	−0.0857	−0.0335

**Table 2:** XRD results of undoped and doped samples.

Sample	Peak Position	d-Spacing	FWHM	Crystallite Size
FVO	8.403	10.514	0.667	126.129
Co	8.305	10.638	0.776	108.324
Mn	8.224	10.743	1.916	43.878
Ni	8.288	10.659	0.726	115.873
Zn	8.329	10.607	0.820	102.595

**Table 3:** Specific surface area of undoped and doped FVO samples.

Sample	FVO	Co	Mn	Ni	Zn
Specific surface area (m^2^g^−1^)	40.13	56.70	88.71	87.96	66.69

## Data Availability

Upon reasonable request, the corresponding author will provide the data reported in this work.

## Supplementary Materials

The following supporting information can be downloaded at: https://www.mdpi.com/article/10.3390/nano14211765/s1, Figure S1: Photographs of synthesized undoped and transition metal-doped FVO samples; Table S1: Ionic radius and electronegativity values of transition metals; Table S2: Gibbs formation energy values of the various transition metal oxides (Ref. [32]); Figure S2: XPS peaks of (a) Fe 2p, (b) V 2p, and (c) O1s for all samples; Figure S3: CV curves at various scan rates and galvanostatic charge–discharge profiles at various current densities, respectively, of (a,f) pristine, (b,g) Co-doped, (c,h) Mn-doped, (d,i) Ni-doped, and (e,j) Zn-doped FVO electrodes; Figure S4: Galvanostatic charge-discharge profiles at various current densities of; (a) pristine (b) Co-doped (c) Mn-doped (d) Ni-doped and (e) Zn-doped; FVO electrodes; Figure S5: EIS data of all electrodes compared; Table S3: k1 and k2 values of electrodes obtained from a plot of *i/v*^1/2^ vs. *v*^1/2^; Figure S6: Contribution ratio of capacitive-controlled process to the capacitance of (a) pristine, (b) Co-doped, (c) Ni-doped, and (d) Zn-doped FVO electrodes.

## References

[1] Chakraborty S., Mary N.L. (2022). Review-an overview on supercapacitors and its applications. J. Electrochem. Soc..

[2] Amedzo-Adore M., Han J.I. (2022). Chemically lithiated layered VOPO_4_ by microwave-assisted hydrothermal method and its electrochemical properties in rechargeable Li-ion batteries and supercapacitor applications. J. Alloy Compd..

[3] Chatterjee D.P., Nandi A.K. (2021). A review on the recent advances in hybrid supercapacitors. J. Mater. Chem. A.

[4] Jalal N.I., Ibrahim R.I., Oudah M.K. (2021). A review on supercapacitors: Types and components. J. Phys. Conf. Ser..

[5] Simon P., Gogotsi Y. (2008). Materials for electrochemical capacitors. Nat. Mater..

[6] Miller J.R., Simon P. (2008). Electrochemical capacitors for energy management. Science.

[7] Huggins R.A. (2000). Supercapacitors and electrochemical pulse sources. Solid State.

[8] Dunn B., Kamath H.J.M. (2000). Tarascon, Electrical energy storage for the grid: A battery of choices. Science.

[9] Yao G., Zhang N., Zhang Y., Zhou T. (2021). Nanostructured transition metal vanadates as electrodes for pseudo-supercapacitors: A review. J. Nanopart. Res..

[10] Ni S., Liu J., Chao D., Mai L. (2019). Vanadate-based materials for Li-ion batteries: The search for anodes for practical applications. Adv. Energy Mater..

[11] Low W.H., Khiew P.S., Lim S.S., Siong C.W., Ezeigwe E.R. (2019). Recent development of mixed transition metal oxide and graphene/mixed transition metal oxide based hybrid nanostructures for advanced supercapacitors. J. Alloys Compd..

[12] Liu M.C., Kong L.B., Kang L., Li X., Walsh F.C., Xing M., Lu C., Ma X.J., Luo Y.C. (2014). Synthesis and characterization of M_3_V_2_O_8_ (M = Ni or Co) based nanostructures: A new family of high performance pseudocapacitive materials. J. Mater. Chem. A.

[13] Wei Q., Wang Q., Li Q., An Q., Zhao Y., Peng Z., Jiang Y., Tan S., Yan M., Mai L. (2018). Pseudocapacitive layered iron vanadate nanosheets cathode for ultrahigh-rate lithium ion storage. Nano Energy.

[14] Chae M.S., Setiawan D., Kim H.J., Hong S.T. (2021). Layered iron vanadate as a high-capacity cathode material for nonaqueous calcium-ion batteries. Batteries.

[15] Wei Q., Jiang Y., Qian X., Zhang L., Li Q., Tan S., Zhao K., Yang W., An Q., Guo Y. (2018). Sodium ion capacitor using pseudocapacitive layered ferric vanadate nanosheets cathode. iScience.

[16] Si Y., Liu G., Deng C., Liu W., Li H., Tang L. (2017). Facile synthesis and electrochemical properties of amorphous FeVO_4_ as cathode materials for lithium secondary batteries. J. Electroanal. Chem..

[17] Rani B.J., Ravi G., Yuvakkumar R., Kumar P., Hong S.I., Velauthapillai D., Thambidurai M., Dang C. (2020). Ni supported anorthic phase FeVO_4_ nanorods for electrochemical water oxidation. Mater. Lett..

[18] Kaneti Y.V., Liu M., Zhang X., Bu Y., Yuan Y., Jiang X., Yu A. (2016). Synthesis of platinum-decorated iron vanadate nanorods with excellent sensing performance toward n-butylamine. Sens. Actuators B.

[19] Amedzo-Adore M., Han J.I. (2023). All layered iron vanadate (FeV_3_O_9_.2.1H_2_O as electrode for symmetric supercapacitor application in aqueous electrolyte. J. Alloy Compd..

[20] Yang J.H., Han D.W., Jo M., Song K., Kim Y., Chou S., Liu H.K., Kang Y.M. (2015). Na_3_V_2_(PO_4_)_3_ particles partly embedded in carbon nanofibers with superb kinetics for ultra-high power sodium ion batteries. J. Mater. Chem. A.

[21] Li W., Han C., Xia Q., Zhang K., Chou S., Kang Y.M., Wang J., Liu H.K., Dou S.X. (2018). Remarkable enhancement in sodium-ion kinetics of NaFe_2_(CN)_6_ by chemical bonding with graphene. Small Methods.

[22] Yang Z., Li G., Sun J., Xie L., Jiang Y., Huang Y., Chen S. (2020). High performance cathode material based on Na_3_V_2_(PO_4_)_2_F_3_ and Na_3_V_2_(PO_4_)_3_ for sodium-ion batteries. Energy Stor. Mater..

[23] Mishra A., Bera G., Mal P., Padmaja G., Sen P., Das P., Chakraborty B., Turpu G.R. (2019). Comparative electrochemical analysis of rGO-FeVO_4_ nanocomposite and FeVO_4_ for supercapacitor application. Appl. Surface Sci..

[24] Zhang K., Kim D., Hu Z., Park M., Noh G., Yang Y., Zhang J., Lau V.W., Chou S., Cho M. (2019). Manganese based layered oxides with modulated electronic and thermodynamic properties for sodium ion batteries. Nat. Commun..

[25] Yousefipour K., Sarraf-Mamoory R., Yourdkhani A. (2019). Iron-doped as an effective strategy to enhance supercapacitive properties of nickel molybdate. Electrochim. Acta.

[26] Dong R., Ye Q., Kuang L., Lu X., Zhang Y., Zhang X., Tan G., Wen Y., Wang F. (2013). Enhanced supercapacitor performance of Mn_3_O_4_ nanocrystals by doping transition-metal ions. ACS Appl. Mater. Interfaces.

[27] Fengxia F., Ruixin Z., Tin Z., Haoyang X., Xiaojuan W., Xinxiang W., Guilei T., Shuhan W., Chenrui Z., Wei X. (2023). Cation-ordered Ni-rich positive electrode material with superior chemical and structural stability enabled by atomic substitution for lithium-ion batteries. Chem. Eng. J..

[28] Chenrui Z., Ruixin Z., Fengxia F., Xinxiang W., Guilei T., Sheng L., Pengfei L., Chuan W., Shuhan W., Chaozhu S. (2024). Phase compatible surface engineering to boost the cycling stability of single-crystalline Ni-rich cathode for high energy density lithium-ion batteries. Energy Storage Mater..

[29] Chenrui Z., Fengxia F., Ruixin Z., Xinxiang W., Guilei T., Sheng L., Pengfei L., Chuan W., Shuhan W., Chaozhu S. (2024). Structural and charge regulation strategy enabling superior cycling stability of Ni-rich cathode materials. ACS Appl. Mater. Interfaces.

[30] Sun C., Luo X. (2022). Tuning the magnetic and electronic properties of monolayer VI_3_ by 3d transition metal doping: A first-principles study. Appl. Surf. Sci..

[31] Chu M., Huang Z., Zhang T., Wang R., Shao T., Wang C., Zhu W., He L., Chen J., Zhao W. (2021). Enhancing the electrochemical performance and structural stability of Ni-rich layered cathode materials via dual-site doping. ACS Appl. Mater. Interfaces.

[32] Jian Z., Feiteng W., Qingqing C., Guoliang W., Lushan M., Chi C., Lijun Y., Zhiqing Z., Daiqian X., Hui Y. (2020). Cobalt/zing dual-sites coordinated with nitrogen in nanofibers enabling efficient and durable oxygen reduction reaction in acidic fuel cells. J. Mater. Chem. A.

[33] Shunmugapriya B., Rose A., Maiyalagan T., Vijayakumar T. (2020). Effect of cobalt doping on the electrochemical performance of trimanganese tetraoxide. Nanotechnology.

[34] Zhang X., Yue L., Zhang S., Feng Y., An L., Wang M., Mi J. (2020). Nickel-doped cobalt molybdate nanorods with excellent cycle stability for aqueous asymmetric supercapacitor. Int. J. Hydrogen Energ..

[35] Zhu X., Wei Z., Ma L., Liang J., Zhang X. (2020). Synthesis and electrochemical properties of Co-doped ZnMn_2_O_4_ hollow nanospheres. Bull. Mater. Sci..

[36] Zhang P., Wang Y., Lin M., Zhang D., Ren X., Yuan Q. (2012). Doping effect of Nb^5+^ on the microstructure and defects of LiFePO_4_. J. Electrochem. Soc..

[37] Li K., Shao J., Xue D. (2013). Site selectivity in doped polyanion cathode materials for Li-ion batteries. Funct. Mater. Lett..

[38] Dean J.A. (1999). Lange’s Handbook of Chemistry.

[39] Li K., Xue D. (2006). Estimation of electronegativity values of elements in different valence states. J. Phys. Chem. A.

[40] Ankinovich E.A., Bekenova G.K., Podlipaeva N.I. (1989). A new hydrous ferrovanadian mineral kazakhstanite Fe_5_^3+^V_3_^4+^V_12_^5+^O_39_(OH)_9_.8.55H_2_O from a carbonaceious-siliceous formation in NW karatau (South Kazakhstan). Zap. Vses. Mineral. Obshch..

[41] Poizot P., Laruelle S., Touboul M., Tarascon J.M. (2003). Wet-chemical synthesis of various iron (III) vanadates (V) by co-precipitation route. C.R. Chim..

[42] Shin J., Yang J., Sergey C., Song M.S., Kang Y.M. (2017). Carbon nanofibers heavy laden with Li_3_V_2_(PO_4_)_3_ particles featuring superb kinetics for high-power lithium ion battery. Adv. Sci..

[43] Amedzo-Adore M., Yang J.H., Han D., Chen M., Agyeman D.A., Zhang J., Zhao R., Kang Y.M. (2021). Oxygen-deficient P2-Na_0.7_Mn_0.75_Ni_0.25_O_2-x_ cathode by a reductive NH_4_HF_2_ treatment for highly reversible Na-ion storage. ACS Appl. Energy Mater..

[44] Park J.S., Kim J., Park W.B., Sun Y.K., Myung S.T. (2017). Effect of Mn in Li_3_V_2-x_Mn_x_(PO_4_)_3_ as high capacity cathodes for lithium batteries. ACS Appl. Mater. Interfaces.

[45] Amedzo-Adore M., Han J.I. (2022). Investigating the pseudo-capacitive properties of interlayer engineered VOPO_4_ by organic molecule intercalation. Ceram. Int..

