# Supplementary material for: Transition Metal-Doped Layered Iron Vanadate (FeV3-xMxO9.2.6H2O, M = Co, Mn, Ni, and Zn) for Enhanced Energy Storage Properties
Source: Nanomaterials (Basel). 2024 Nov 3;14(21):1765. doi: 10.3390/nano14211765 (PMC11547368; doi:10.3390/nano14211765)
Supplement: Supplementary file 1 [file nanomaterials-14-01765-s001.zip › nanomaterials-3271859-supplementary.pdf]

nanomaterials

MDPI

Supplementary information

Transition Metal-Doped Layered Iron Vanadate ($FeV_{3-x}M_xO_9.2.6H_2O$, M = Co, Mn, Ni, and Zn) for Enhanced Energy Storage Properties

Mawuse Amedzo-Adore and Jeong In Han *

Department of Chemical and Biochemical Engineering, Dongguk University, Seoul 04620, Republic of Korea; mendeladore@dongguk.edu

* Correspondence: hanji@dongguk.edu

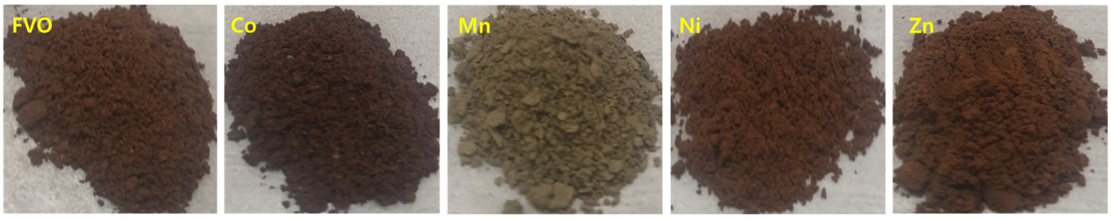

Figure S1. Photographs of synthesized undoped and transition metal-doped FVO samples.

Table S1: Ionic radius and electronegativity values of transition metals

Ions	Fe^{3+}	V^{4+}	Co^{2+}	Mn^{2+}	Ni^{2+}	Zn^{2+}
Ionic radius (nm)	0.069	0.072	0.079	0.081	0.083	0.088
Electroneg-ativity	1.556	1.795	1.321	1.263	1.367	1.336

Table S2: Gibbs formation energy values of the various transition metal oxides (Ref. 32)

Ions	V^{4+}	Co^{2+}	Mn^{2+}	Ni^{2+}	Zn^{2+}
Gibbs formation energy (kJ mol^{-1})	−717.6	−214	−362.9	−211.7	−320.5

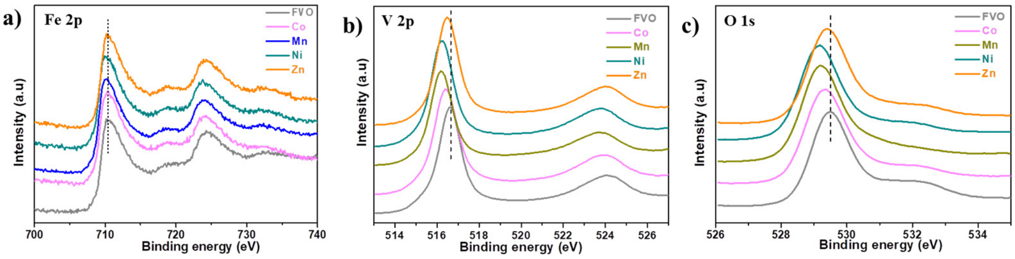

Figure S2. XPS peaks of **(a)** Fe 2p, **(b)** V 2p and **(c)** O1s; for all samples.

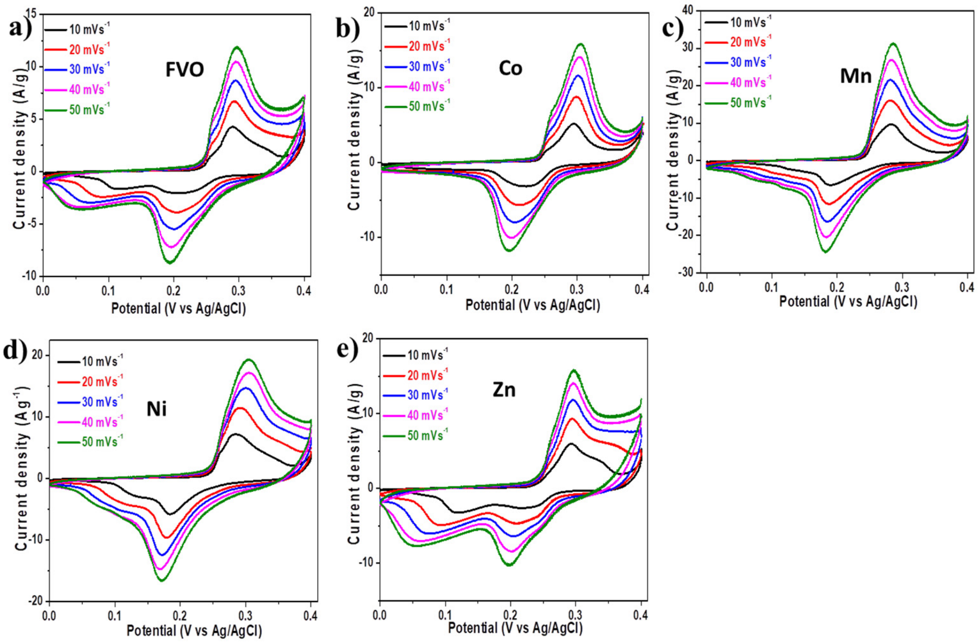

Figure S3. CV curves at various scan rates of; **(a)** pristine **(b)** Co-doped **(c)** Mn-doped **(d)** Ni-doped and **(e)** Zn-doped; FVO electrodes.

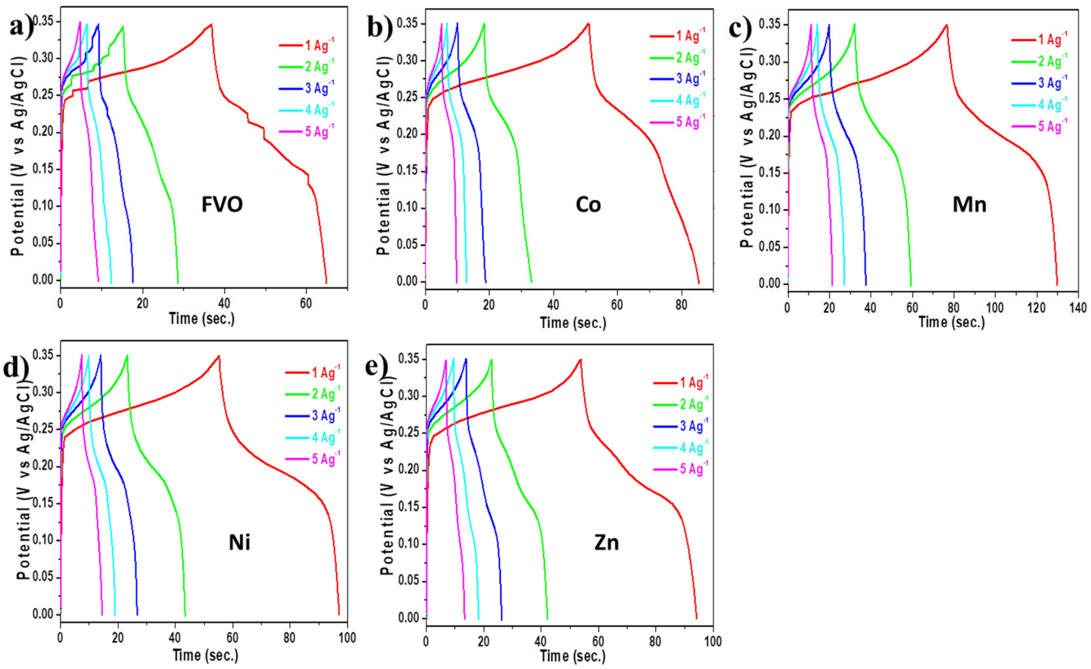

Figure S4. Galvanostatic charge-discharge profiles at various current densities of; **(a)** pristine **(b)** Co-doped **(c)** Mn-doped **(d)** Ni-doped and **(e)** Zn-doped; FVO electrodes.

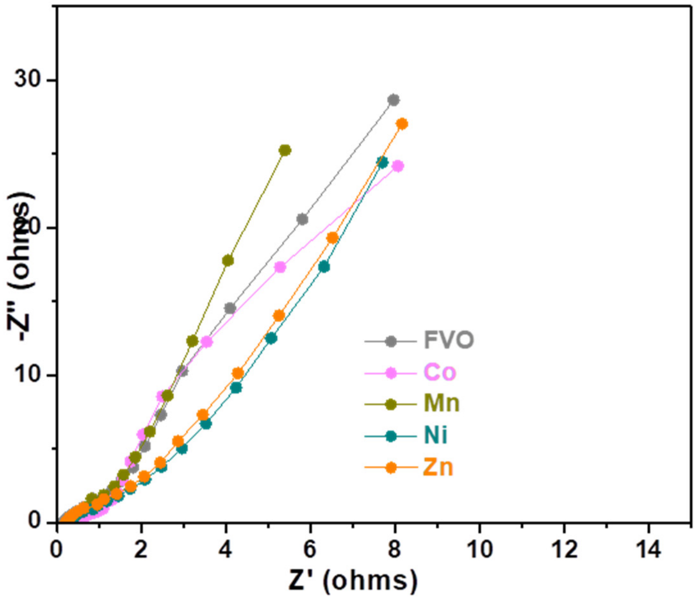

Figure S5. EIS data of all electrodes compared.

Table S3: k1 and k2 values of electrodes obtained from a plot of $i/v^{1/2}$ vs $v^{1/2}$

Electrode	FVO	Co	Mn	Ni	Zn
k1	0.0769	0.0811	0.3407	0.1543	0.1052
k2	1.6518	1.0877	1.9755	1.1805	1.9538

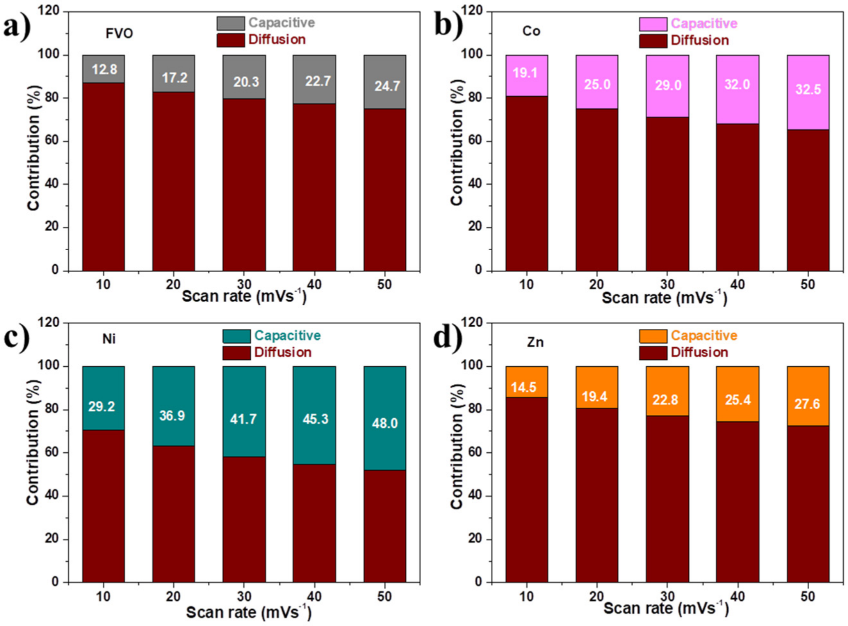

Figure S6. Contribution ratio of capacitive-controlled process to the capacitance of **(a)** pristine **(b)** Co-doped **(c)** Ni-doped **(d)** Zn-doped; FVO electrodes.
